# Expression and in vitro assessment of tumorigenicity for NOD1 and NOD2 receptors in breast cancer cell lines

**DOI:** 10.1186/s13104-018-3335-4

**Published:** 2018-04-03

**Authors:** Fernando J. Velloso, Mari Cleide Sogayar, Ricardo G. Correa

**Affiliations:** 1 Cell and Molecular Therapy Center (NUCEL-NETCEM), Internal Medicine Department, School of Medicine, University of São Paulo (USP), São Paulo, SP 05360-130 Brazil; 2 Sanford Burnham Prebys Medical Discovery Institute, 10901 North Torrey Pines Rd., La Jolla, CA 92037 USA

**Keywords:** NOD1, NOD2, NLR, Hs578T, Breast cancer, γ-Tri-DAP, MDP

## Abstract

**Objective:**

Immune-related pathways have been frequently associated to tumorigenesis. NOD1 and NOD2 are innate immune receptors responsible for sensing a subset of bacterial-derived components, and to further translate these pathogenic signals through pro-inflammatory and survival pathways. NOD1 and NOD2 have been further associated with tumorigenesis, particularly in gastrointestinal cancers. *NOD1* has also been suggested to be a tumor suppressor gene in a model of estrogen receptor-dependent breast cancer. Contrarily, *NOD2* polymorphisms are associated with higher risk of breast cancer, with no tumor suppressor role being reported. To better delineate this issue, we investigated *NOD1* and *NOD2* expression in a panel of breast cancer cell lines, as well as their potential impact in breast tumorigenesis based on in vitro assays.

**Results:**

The highly invasive Hs578T breast cell line presented the second highest *NOD1* expression and the lowest *NOD2* expression in our panel. Therefore, we investigated whether NOD1 and/or NOD2 might act as a tumor suppressor in this cell model. Our studies indicate that overexpression of either *NOD1* or *NOD2* reduces cell proliferation and increases clonogenic potential in vitro. Elucidation of NOD1 and NOD2 effects on tumor cell viability and proliferation may unveil potential targets for future therapeutic intervention.

## Introduction

Breast cancer is the malignancy with the highest incidence in women worldwide, accounting for 29% of all diagnosed cancers in females [1]. Despite clinical improvements in diagnosis and treatment, breast cancer remains the leading cause of cancer mortality among women, representing 14% of all deaths from cancer in women [2], mostly associated to metastatic tumors [3].

Breast cancer is classified according to immunohistological detection of protein markers, including receptors for estrogen (ER), progesterone (PR), androgen (AR), and the amplified HER2 (Human Epidermal Growth Factor Receptor 2) receptor [4]. Approximately 15% of all breast tumors lack expression of ER, PR and amplification of HER2, being therefore classified as triple negative breast cancers (TNBC). The absence of well-defined molecular targets, such as ERα and PR, prevents the use of selective drug therapies, rendering TNBC the most lethal type of breast cancer [5, 6].

Inflammation is an important underlying factor for cancer development [7]. In a number of tissues, including breast, tumor onset and progression have been associated to immune-related molecules, such as interleukins, caspases and a set of cytosolic receptors called NLRs (NACHT and Leucine Rich Repeat domain containing proteins). NLRs are pattern recognition receptors (PRRs) which recognize both pathogen-associated molecular patterns (PAMPs) and danger associated molecular patterns (DAMPs), thus acting as innate immunity “sensors” towards pathogen-derived components and cellular damage/stress. NOD1 and NOD2 (nucleotide-binding oligomerization domain-containing protein 1 and 2) are two major NLRs that directly modulate pro-inflammatory pathways, including NF-κB and MAPK [8]. NOD1 and NOD2 display variable tandem C-terminal leucine-rich repeat domains (LRRs), which are responsible for ligand recognition and allow these receptors to detect the bacterial peptidoglycan iE-DAP (gamma-d-glutamyl-meso-diaminopimelic acid) and MDP (muramyl dipeptide), respectively [8]. Ligand-bound NOD1 and NOD2 recruit RIP2, which activates the IKK complex towards NF-κB and stress kinase cascades through MAPKs [9].

Persistent activation and deficiency of NOD1 and NOD2 receptors have been associated to gastrointestinal cancers [10]. In other tissues, including breast, *NOD1* and *NOD2* knockdown models display increased predisposition for tumorigenesis [11]. Additionally, *NOD1* and *NOD2* polymorphisms have been associated to increased risk for several cancer types, including breast [12, 13]. In the estrogen-dependent MCF7 breast cancer cell line, NOD1 activation was shown to promote RIP2 and caspase 8-mediated apoptosis and to reduce estrogen-induced proliferative responses in vitro [14]. Likewise, the absence of NOD1 leads to increased sensitivity to estrogen-induced cell proliferation and a failure to undergo NOD1-dependent apoptosis. Upon injection into a severe combined immune deficiency (SCID) mouse xenograft model, MCF7 cells lacking NOD1 displayed increased estrogen-dependent tumor growth [15]. Moreover, *NOD1* overexpression halted estrogen-dependent tumor proliferation. Therefore, *NOD1* has been proposed to act as a tumor suppressor gene in ER-positive cells. Contrarily, NOD2 activation does not induce apoptosis in this cell line [14, 15].

In order to determine whether NOD1 and/or NOD2 play significant roles in the onset and progression of breast cancer, here we evaluated the expression of *NOD1* and *NOD2* in a panel of progressively invasive breast cancer-derived cell lineages. In addition, we analyzed the impact of *NOD1* and *NOD2* overexpression in breast cancer, based on cell proliferation and clonogenic assays.

## Main text

### Methods

#### *NOD1* and *NOD2* expression profiling in breast cancer derived cell lines

Expression profiling was obtained for a panel of breast cancer derived cell lines, including non-tumorigenic MCF10A (ATCC^®^: CRL-10317™; ER−/PR−/AR−/HER2−) and MCF12A (ATCC^®^: CRL-10782™; ER−/PR−/AR+/HER2−), estrogen-positive MCF-7 (ATCC^®^ HTB-22™; ER+/PR+/AR+/HER2−) and ZR-75-1 (ATCC^®^ CRL-1500™; ER+/PR+/AR+/HER2+), and estrogen-negative SK-BR-3 (ATCC^®^ HTB-30™; ER−/PR−/AR+/HER2+), MDA-MB-231 (ATCC^®^ HTB-26™; ER−/PR−/AR+/HER2−) and Hs578T (ATCC^®^ HTB-126™; ER−/PR−/AR+/HER2−). Cells lines were obtained from ATCC (American Type Culture Collection) and analyzed at low passages (3–6) to avoid genetic drift aberrations. Replicated experiments were carried out with cells at increasing sequential passages.

Total RNA was isolated using Trizol reagent (Thermo), followed by DNAse I treatment (Thermo) for 20 min at 37 °C. Reverse transcription was performed using Superscript III polymerase (Thermo), according to manufacturer’s protocols. RT-qPCR was carried out using FAST SYBR Green Master Mix (Thermo) in a ViiA 7 Real-Time PCR System (Thermo). Transcript amount quantification was calculated using the Comparative CT Method (ΔΔCt; [16]), based on three technical replicates, with the QuantStudio™ Software V1.3 (Thermo). Graph design and statistical analyses were performed in Graphpad Prism V6 (Graphpad Software). Primers for the following human genes were synthesized (IDT): *NOD1* (forward: 5′-CTGCTCACTCAGAGCAAAGTCGT-3′; reverse: 5′-GTCCATGTAGATCTCCTCCAGCA-3′), *NOD2* (forward: 5′-AAATCAGGTTGCCGATCTTCA-3′; reverse: 5′-CAGCCAATCCATTCGCTTTC-3′), *RPL13A* (forward: 5′-CCTGGAGGAGAAGAGGAAAGAGA-3′; reverse: 5′-TTGAGGACCTCTGTGTATTTGTCAA-3′), *HMBS* (forward: 5′-TGGACCTGGTTGTTCACTCCTT-3′; reverse: 5′-CAACAGCATCATGAGGGTTTTC-3′).

#### Overexpression of *NOD1* and *NOD2* in Hs578T breast cancer cells

*NOD1* and *NOD2* cDNAs were previously subcloned into a lentiviral 6xHis-FLAG-containing vector, co-expressing *EGFP* under an IRES sequence [17]. Lentivirus production followed as previously described [18]. Hs578T cells were transduced by spinfection (MOI > 5). Transduced GFP-positive cells were sorted using a FACS Aria II flow cytometer (BD Biosciences).

#### Western blotting

Ectopic proteins were detected by immunoblotting using mouse monoclonal anti-FLAG antibody (ab18230, Abcam). Reversible Ponceau staining was used to control the equal loading of protein lysates.

#### Growth curves

5 × 10^3^ cells from each population were cultured in 3.8 cm^2^ wells (12-well plate) for 6 days. Cells were harvested every 24 h and the total cell number was obtained using the Accuri C6 Plus flow cytometry system (BD Biosciences). Statistical analysis was carried out using Graphpad Prism V6 (Graphpad Software), with two-way repeated measures ANOVA test (n = 3; 0.05 alpha), querying the cell populations as the source of variation.

Population doubling time (PDT), was calculated from the equation Δt × [ln2/(lnNt − lnN0)], where Δt is the duration of cell proliferation (exponential phase) in hours, and N0 and Nt are the respective numbers of cells at the beginning and end of this period [19, 20].

#### Colony formation assay in solid substrate

2 × 10^2^ cells from each population were cultured in 9.5 cm^2^ wells (6-well plate) for 12 days. Colonies were counted after fixation (4% formaldehyde; Sigma Aldrich) and staining (0.05% crystal violet). Statistical analysis was performed using two-tailed, unpaired, student’s t-test (n = 3; 0.05 alpha) (Graphpad Prism V6).

#### Colony formation assay in soft-agar substrate

1 × 10^4^ cells from each population were seeded in 1.9 cm^2^ wells (24-well plate) previously covered with 0.5 mL DMEM growth medium containing 0.6% agar. After seeding, a layer of 0.5 mL 0.3% agar DMEM was added and allowed to gellify before addition of 0.5 mL DMEM per well. Cultures were maintained for 14 days. Colonies were fixed in 4% formaldehyde and counted. Statistical analysis was performed using two-tailed, unpaired, student’s t-test (n = 4; 0.05 alpha) (Graphpad Prism V6).

### Results

#### *NOD1* and *NOD2* are differentially expressed in breast cancer cell lines

**Fig. 1:**
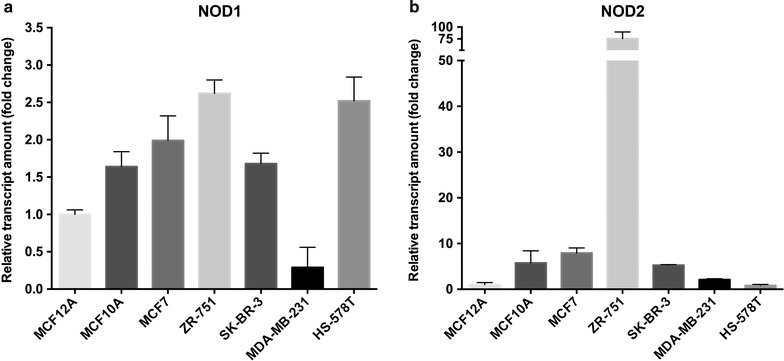
*NOD1* and *NOD2* expression in breast cancer cells. Relative mRNA quantitation was performed by RT-qPCR, using a panel of breast cancer cell lineages. Values are presented as fold change relative to expression in the non-tumorigenic MCF12A cell line, after normalization using *RPL13A* and *HMBS* as endogenous controls (mean + standard deviation, n = 3)

Figure 1 shows the expression profiles of *NOD1* and *NOD2* genes in a panel of breast cancer-derived cell lines, including estrogen-positive (MCF-7 and ZR-75-1) and estrogen-negative (MCF10A, MCF12A, SK-BR-3, MDA-MB-231 and Hs578T) cell lines. We found that *NOD1* and *NOD2* expression varies among these cell lines, with no clear pattern discriminating estrogen receptor-positive or negative groups. Interestingly, the Hs578T cell line presented the highest expression of *NOD1* and the lowest expression of *NOD2* in our panel. Due to its specific characteristics, namely, its tumorigenic potential and TNBC origin [21], we decided to overexpress *NOD1* or *NOD2* in Hs578T cells for further functional studies.

#### Overexpression status of *NOD1/2* in transduced Hs578T cells

**Fig. 2:**
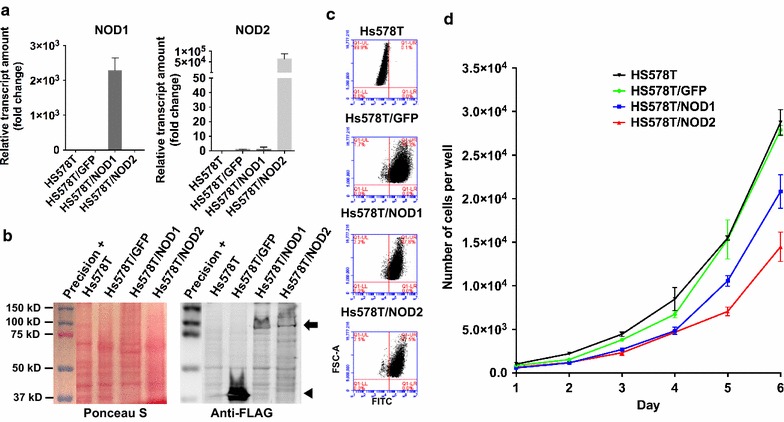
Hs578T/NOD1 and Hs578T/NOD2 populations highly express respective transgenes and display a lower proliferative rate. **a** Relative *NOD1* and *NOD2* transcripts detection by RT-qPCR in Hs578T cell populations after transduction with lentiviruses for overexpression of *GFP*, *NOD1* or *NOD2*. Normalization was performed as indicated in Fig. 1 (mean + standard deviation, n = 3). **b** Ponceau S staining and anti-FLAG immunoblot detection of overexpressed GFP (arrowhead), NOD1 and NOD2 (arrow) proteins in transduced H578T populations. **c** Detection of GFP-positive cells in Hs578T populations overexpressing *NOD* genes by flow cytometry (FSC-A vs FITC). Positive cells are gated inside the upper right quadrant, representing over 97% of all transduced populations. **d** Growth curves comparing the proliferative rate of the wild-type versus transduced Hs578T populations overexpressing *GFP*, *NOD1* or *NOD2*. 5x10^3^ cells were seeded in 3.8 cm^2^ wells (12-well plate) and cultured for six days. Total cell number per well is presented at each time point (two-way ANOVA, n=3)

Hs578T/NOD1 and Hs578T/NOD2 populations were generated, overexpressing high amounts of ectopic NOD1 and NOD2 at both mRNA and protein levels (Fig. 2a, b). A control population, expressing only *GFP*, displayed *NOD1* and *NOD2* expression levels similar to those of the wild-type  Hs578T cell line (Fig. 2a). Since Hs578T/NOD cells co-express *GFP* [17, 18], we used flow cytometry to ensure over 97% enrichment in GFP-positive cell content (Fig. 2c).

#### Hs578T/NOD1 and Hs578T/NOD2 populations display a lower proliferation rate

In vitro assays were carried out, with cell populations overexpressing *NOD1*/*2*, to assess their proliferative potential and viability. Hs578T/NOD1 and Hs578T/NOD2 displayed decreased proliferative rates when compared to wild-type (Hs578T) and GFP-only control cells (two-way ANOVA, P ≤ 0.005, n = 3). Control populations (wild-type and GFP-transduced cells) displayed statistically identical proliferative rates, with doubling times of 24.9 and 23.9 h, respectively. Intriguingly, the Hs578T/NOD2 population displayed the lowest growth rate, even when compared to Hs578T/NOD1 cells (33.4 vs. 28.9 h, respectively) (Fig. 2d).

#### Hs578T/NOD1 and Hs578T/NOD2 cell populations display higher cellular viability

**Fig. 3:**
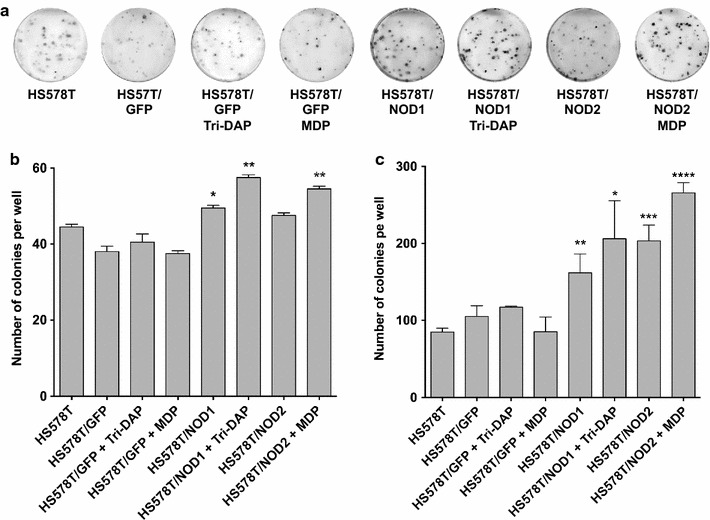
Hs578T/NOD1 and Hs578T/NOD2 cell populations display higher viability in solid substrate and in soft agarose, and this effect is enhanced by NOD1/2 agonists. Colony formation assays, comparing the total number of colonies per well, from wild-type or transduced Hs578T populations overexpressing *GFP*, *NOD1* or *NOD2.* **a** Images of colonies formed by the different Hs578T populations in solid substrate upon staining with crystal violet. **b** Total number of colonies formed per well in both **b** solid and **c** soft agarose substrates, by transduced Hs578T populations in comparison to the wild-type. In both assays, Hs578T/NOD1 and Hs578T/NOD2 populations were treated with 5 μg/mL γ-tri-DAP and MDP (Invivogen), respectively (unpaired student’s t-test; *P ≤ 0.05; **P ≤ 0.01; ***P ≤ 0.001; ****P ≤ 0.0001. **a** n = 3; **b** n = 4)

We employed in vitro colony formation assays to infer tumor growth and viability of the *NOD1*/*2*-overexpressing populations. Upon seeding on solid substrate, the control populations presented statistically similar number of colonies per well. However, Hs578T/NOD1 cells showed an increased number of colonies formed per well (unpaired student’s t-test; P < 0.05; n = 3) when compared to the controls. This effect was further enhanced by treatment with 5 μg/mL γ-tri-DAP, a NOD1-specific agonist (unpaired student’s t-test; P ≤ 0.01; n = 3). Hs578T/NOD2 displayed a non-statistical tendency for increased number of colonies in the absence of any treatment, and a statistically significant increase in colony number upon treatment with 5 μg/mL MDP, a NOD2-specific agonist (unpaired student’s t-test; P ≤ 0.01; n = 3) (Fig. 3a, b).

#### Hs578T/NOD1 and Hs578T/NOD2 display higher anchorage-independent growth

The ability to form colonies in soft agar, a close in vitro approximation of the in vivo tumorigenic potential, was also investigated. As shown in Fig. 3c, both Hs578T/NOD1 and Hs578T/NOD2 cells displayed an increased number of colonies in semi-solid substrate (unpaired student’s t-test; P < 0.01; n = 4; unpaired student's t-test; P ≤ 0.001; n = 4). Again, this effect was further enhanced by treatment with 5 μg/mL γ-tri-DAP (unpaired student's t-test; P ≤ 0.05; n = 4) or 5 μg/mL MDP (unpaired student's t-test; P ≤ 0.0001; n = 4) (Fig. 3c).

### Discussion

Here, we show that *NOD1* and *NOD2* display variable expression levels in a panel of estrogen receptor-positive and estrogen receptor-negative breast cancer cell lines. NOD1 was previously described as a possible tumor suppressor gene in an estrogen receptor-positive cellular model, acting through the blockage of ERα [15]. We elected the triple-negative Hs578T cell line to investigate whether the same effect would be observed in an estrogen receptor-negative cell line, by generating transduced cells overexpressing NOD1 or NOD2 receptors. In the aforementioned report on the estrogen receptor-positive model, NOD1 overexpression had no effect on cell proliferation, whereas silencing of this receptor actually increased the estrogen-dependent cell proliferation. In our model, the *NOD*1 overexpressing cells displayed a decreased proliferative rate in vitro. Remarkably, *NOD2* overexpression caused an even more pronounced decrease in cell proliferation. Therefore, our data suggest that both NOD1 and NOD2 signals may regulate cell proliferation in this TNBC model, through a presumptive ER-independent pathway.

Our in vitro colony formation assays indicated an increased viability and cell growth rate in *NOD1*- and *NOD2*-overexpressing Hs578T populations. An even more pronounced effect was found for NOD2 towards cell proliferation and colony formation. The impact of *NOD1*/*2* overexpression in colony formation was also increased by specific agonists, further indicating that activation of these receptors is directly linked to the effects observed. The apparent contradiction between proliferation and colony formation in our model may be explained by the variety of signals triggered by NOD receptors in downstream pathways. The effects observed in cell proliferation may be a direct result of signals towards MAPK pathway, thus modulating cell cycle checkpoints. On the other hand, colony formation may be affected by signaling pathways related to NF-κB, increasing cellular viability through apoptotic escape or promoting independence from contact and anchorage signals such as ECAD (E-cadherin) [22], ICAM1 (Intercellular adhesion molecule 1) [23] and VCAM1 (Vascular cell adhesion Molecule 1) [24].

Given the incidence and high mortality rate of TNBC, decoding sub-pathways through which NOD receptors modulate cell proliferation may offer potential new targets for future therapeutic interventions.

## Limitations

A more conclusive assessment of NOD1/2 levels could benefit from western blot detection. However, due to their typically limited protein levels in a vast number of cell models, endogenous *NOD1* and *NOD2* amounts are usually evaluated by RT-qPCR [15, 17, 25, 26]. As an example, immunoblot detection of NOD1 in HCT-116 cells, a bonafide cell model for NOD1/2 signaling, can only be achieved after immunoprecipitation and enrichment with a second NOD1 specific antibody [17].

Additionally, generating Hs578T populations with null expression of *NOD1* and/or *NOD2* would provide a valuable functional corroboration model to complement our overexpression results. Also, determining the sensitivity to apoptotic induction in our overexpressing populations could allow a better understanding of the pathways participating in the cellular effects observed.

## Abbreviations

AR: Androgen receptor
DAMP: Danger-associated molecular patterns
ECAD: E-cadherin
ER: Estrogen receptor
ERα: Estrogen receptor alpha
HER2: Human epidermal growth factor receptor 2
iE-DAP: Gamma-d-glutamyl-meso-diaminopimelic acid
IKK: Inhibitor of κB (IκB) kinase
LRR: Leucine-rich repeat domain
MAPK: Mitogen-activated protein kinase
MDP: Muramyl dipeptide
NLR: NACHT and Leucine-Rich Repeat domain containing protein
NOD1: Nucleotide-binding Oligomerization Domain-containing protein 1
NOD2: Nucleotide-binding Oligomerization Domain-containing protein 2
PAMP: Pathogen-associated molecular pattern
PR: Progesterone receptor
PRR: Pattern recognition receptor
SCID: Severe combined immune deficiency
TNBC: Triple negative breast cancer
γ-tri-DAP: l-Ala-y-d-Glu-mDAP
VCAM1: Vascular cell adhesion molecule 1
